# Supplementary material for: Supporting care engagement in primary care; the development of a maturity matrix
Source: PLoS One. 2023 Jan 5;18(1):e0279542. doi: 10.1371/journal.pone.0279542 (PMC9815637; doi:10.1371/journal.pone.0279542)
Supplement: S1 File — (PDF) [file pone.0279542.s001.pdf]

S1 File. Scoping literature search

Table 1 Database searching and terms		
**Databases searched**	Search terms and search strategy	Number of hits
**OVID MEDLINE ALL**	Exploded (MESH)terms	
**EMBASE**	1. Patient Participation	45,276
**PsycINFO**	2. Patient Engagement (only MEDLINE)	21,728
	3. Patient Empowerment (only MEDLINE)	21,728
	4. Ambulatory Care	96,953
	5. Primary Health Care	283,425
	6. Family Practice (only MEDLINE and EMBASE)	134,288
	Keyword in a multipurpose set of fields (mp. = ti, ab, hw, tn, ot, dm, mf, dv, kw, fx, dq, nm, kf, ox, px, rx, ui, sy, tc, id, tm, mh)	
	7. Patient Participation	50,583
	8. Patient Engagement	3,396
	9. Patient Empowerment	2,387
	10. Ambulatory Care	102,708
	11. Primary Care	236,773
	12. Family Practice	81,752
	Combining terms	
	13. #1 OR #2 OR #3 OR #7 OR #8 OR #9	56,084
	14. #4 OR #5 OR #6 OR #10 OR #11 OR #12	651,278
	Initial selection of studies	
	15. #13 AND #14	6,442
	16. Limit #15 to year= "2001-2016"	5,261
	17. Limit #16 to abstracts	4,397
	18. Limit #17 to English language	4,232
	After deduplication in Endnote	3,086

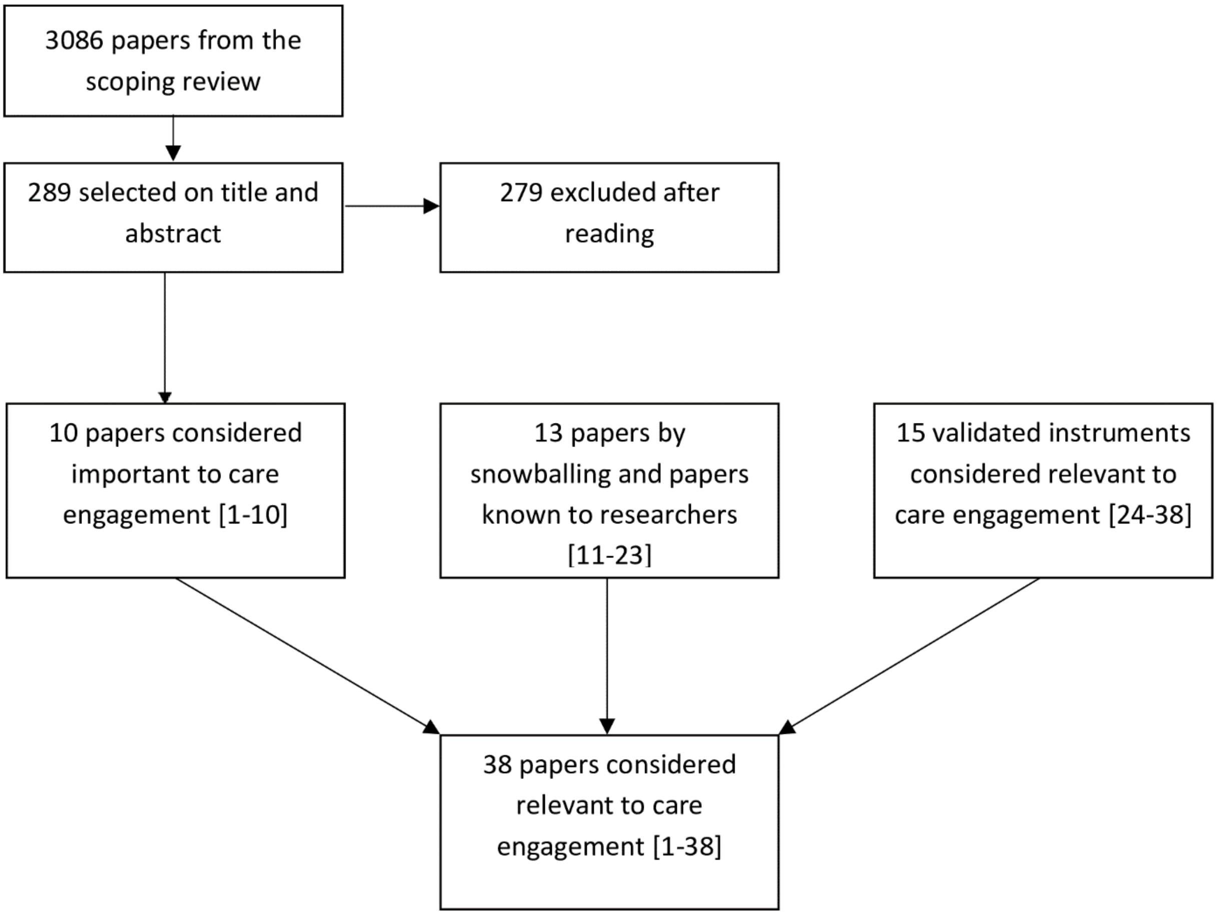
3086 papers from the scoping review
289 selected on title and abstract
279 excluded after reading
10 papers considered important to care engagement [1-10]
13 papers by snowballing and papers known to researchers [11-23]
15 validated instruments considered relevant to care engagement [24-38]
38 papers considered relevant to care engagement [1-38]

References scoping literature search

1. Barry MJ. Shared decision making: informing and involving patients to do the right thing in health care. J Ambul Care Manag. 2012 Apr-Jun;35(2):90-8. doi: 10.1097/JAC.0b013e318249482f
2. Bernabeo E, Holmboe ES. Patients, providers, and systems need to acquire a specific set of competencies to achieve truly patient-centered care. Health Aff (Millwood). 2013 Feb;32(2):250-8. doi: 10.1377/hlthaff.2012.1120
3. Coulter A. Patient engagement--what works? J Ambul Care Manag. 2012 Apr-Jun;35(2):80-9. doi: 10.1097/JAC.0b013e318249e0fd
4. Drenkard K, Swartwout E, Deyo P, O'Neil MB, Jr. Interactive Care Model: A Framework for More Fully Engaging People in Their Healthcare. J Nurs Adm. 2015 Oct;45(10):503-10. doi: 10.1097/NNA.0000000000000242
5. Elwyn G, Edwards A, Wensing M, Hood K, Atwell C, Grol R. Shared decision making: developing the OPTION scale for measuring patient involvement. Qual Saf Health Care. 2003 Apr;12(2):93-9. doi: 10.1136/qhc.12.2.93
6. Elwyn G, Lloyd A, May C, van der Weijden T, Stiggelbout A, Edwards A, et al. Collaborative deliberation: a model for patient care. Patient Educ Couns. 2014 Nov;97(2):158-64. doi: 10.1016/j.pec.2014.07.027
7. Hobbs JL. A dimensional analysis of patient-centered care. Nurs Res. 2009 Jan-Feb;58(1):52-62. doi: 10.1097/NNR.0b013e31818c3e79
8. Jordan JE, Briggs AM, Brand CA, Osborne RH. Enhancing patient engagement in chronic disease self-management support initiatives in Australia: the need for an integrated approach. Med J Aus. 2008 Nov 17;189(S10):S9-S13. doi: 10.5694/j.1326-5377.2008.tb02202.x

9. Osborn R, Squires D. International perspectives on patient engagement: results from the 2011 Commonwealth Fund Survey. J Ambul Care Manag. 2012 Apr-Jun;35(2):118-28. doi: 10.1097/JAC.0b013e31824a579b

10. Wasson JH, Forsberg HH, Lindblad S, Mazowita G, McQuillen K, Nelson EC. The medium is the (health) measure: patient engagement using personal technologies. J Ambul Care Manag. 2012 Apr-Jun;35(2):109-17. doi: 10.1097/JAC.0b013e31824a235e

11. Berwick DM. What 'patient-centered' should mean: confessions of an extremist. Health Aff (Millwood). 2009 Jul-Aug;28(4):w555-65. doi: 10.1377/hlthaff.28.4.w555

12. Dennis SM, Zwar N, Griffiths R, Roland M, Hasan I, Powell Davies G, et al. Chronic disease management in primary care: from evidence to policy. Med J Aust. 2008 Apr;188(S8):S53-6. doi: 10.5694/j.1326-5377.2008.tb01745.x

13. Elwyn G, Frosch DL, Kobrin S. Implementing shared decision-making: consider all the consequences. Implement Sci. 2016 Aug 8;11:114. doi: 10.1186/s13012-016-0480-9

14. Frosch DL, Elwyn G. Don't blame patients, engage them: transforming health systems to address health literacy. J Health Commun. 2014 Oct;19 Suppl 2:10-4. doi: 10.1080/10810730.2014.950548

15. Gagliardi AR, Legare F, Brouwers MC, Webster F, Badley E, Straus S. Patient-mediated knowledge translation (PKT) interventions for clinical encounters: a systematic review. Implement Sci. 2016 Feb 29;11:26. doi: 10.1186/s13012-016-0389-3

16. Gruman J, Rovner MH, French ME, Jeffress D, Sofaer S, Shaller D, et al. From patient education to patient engagement: implications for the field of patient education. Patient Educ Couns. 2010 Mar;78(3):350-6. doi: 10.1016/j.pec.2010.02.002

17. Hibbard J, Lorig K. The dos and don'ts of patient engagement in busy office practices. J Ambul Care Manag. 2012 Apr-Jun;35(2):129-32. doi: 10.1097/JAC.0b013e3182496647

18. Hibbard JH. Engaging health care consumers to improve the quality of care. Med Care. 2003 Jan;41(1 Suppl):I61-70. doi: 10.1097/00005650-200301001-00007

19. Joseph-Williams N, Elwyn G, Edwards A. Knowledge is not power for patients: a systematic review and thematic synthesis of patient-reported barriers and facilitators to shared decision making. Patient Educ Couns. 2014 Mar;94(3):291-309. doi: 10.1016/j.pec.2013.10.031

20. Kinnersley P, Edwards A, Hood K, Cadbury N, Ryan R, Prout H, et al. Interventions before consultations for helping patients address their information needs. Cochrane Database Syst Rev. 2007 Jul 18;3(3):CD004565. doi: 10.1002/14651858

21. Legare F, Thompson-Leduc P. Twelve myths about shared decision making. Patient Educ Couns. 2014 Sep;96(3):281-6. doi: 10.1016/j.pec.2014.06.014

22. Legare F, Witteman HO. Shared decision making: examining key elements and barriers to adoption into routine clinical practice. Health Aff (Millwood). 2013 Feb;32(2):276-84. doi: 10.1377/hlthaff.2012.1078

23. Sarrami-Foroushani P, Travaglia J, Debono D, Braithwaite J. Key concepts in consumer and community engagement: a scoping meta-review. BMC Health Serv Res. 2014 Jun 13;14(1):250. doi: 10.1186/1472-6963-14-250

24. Rademakers J, Nijman J, van der Hoek L, Heijmans M, Rijken M. Measuring patient activation in The Netherlands: translation and validation of the American short form Patient Activation Measure (PAM13). BMC Public Health. 2012 Jul 31;12(1):577. doi: 10.1186/1471-2458-12-577

25. Hibbard JH, Stockard J, Mahoney ER, Tusler M. Development of the Patient Activation Measure (PAM): conceptualizing and measuring activation in patients and consumers. Health Serv Res. 2004 Aug;39(4 Pt 1):1005-26. doi: 10.1111/j.1475-6773.2004.00269.x

26. ten Klooster PM, Oostveen JC, Zandbelt LC, Taal E, Drossaert CH, Harmsen EJ, et al. Further validation of the 5-item Perceived Efficacy in Patient-Physician Interactions (PEPPI-5) scale in patients with osteoarthritis. Patient Educ Couns. 2012 Apr;87(1):125-30. doi: 10.1016/j.pec.2011.07.017

27. Maly RC, Frank JC, Marshall GN, DiMatteo MR, Reuben DB. Perceived efficacy in patient-physician interactions (PEPPI): validation of an instrument in older persons. J Am Geriatr Soc. 1998 Jul;46(7):889-94. doi: 10.1111/j.1532-5415.1998.tb02725.x

28. Osborne RH, Elsworth GR, Whitfield K. The Health Education Impact Questionnaire (heiQ): an outcomes and evaluation measure for patient education and self-management interventions for people with chronic conditions. Patient Educ Couns. 2007 May;66(2):192-201. doi: 10.1016/j.pec.2006.12.002

29. Barr PJ, Thompson R, Walsh T, Grande SW, Ozanne EM, Elwyn G. The psychometric properties of CollaboRATE: a fast and frugal patient-reported measure of the shared decision-making process. J Med Internet Res. 2014 Jan 03;16(1):e2. doi: 10.2196/jmir.3085

30. CBO. [Z-scan: a self-assessment instrument on self-management support for healthcare professionals (Dutch)] Utrecht: CBO; 2012. [Access date 2021 Feb 04] Available from: https://zelfzorgondersteund-instrumentenkiezer.nl/wp-content/uploads/2016/03/Z-scan.pdf.

31. Brownson CA, Miller D, Crespo R, Neuner S, Thompson J, Wall JC, et al. A quality improvement tool to assess self-management support in primary care. Jt Comm J Qual Patient Saf. 2007 Jul;33(7):408-16. doi: 10.1016/s1553-7250(07)33047-x

32. Wensing M, van Lieshout J, Jung HP, Hermsen J, Rosemann T. The Patients Assessment Chronic Illness Care (PACIC) questionnaire in The Netherlands: a validation study in rural general practice. BMC Health Serv Res. 2008 Sep 1;8(1):182. doi: 10.1186/1472-6963-8-182

33. Berendsen AJ, Groenier KH, de Jong GM, Meyboom-de Jong B, van der Veen WJ, Dekker J, et al. [Assessment of patient’s experiences across the interface between primary and secondary care: Consumer Quality Index Continuum of Care (Dutch)]. Samenwerking tussen huisarts en specialist (Wat vinden de patiënten en dokters?). Houten: Bohn Stafleu van Loghum; 2008. p. 103-16. . [Access date 2021 Feb 04]. Available from: https://www.rug.nl/research/portal/files/14515671/07emb_h7.pdf

34. Hoerger M, Chapman BP, Mohile SG, Duberstein PR. Development and psychometric evaluation of the Decisional Engagement Scale (DES-10): A patient-reported psychosocial survey for quality cancer care. Psychol Assess. 2016 Sep;28(9):1087-100. doi: 10.1037/pas0000294

35. Ouwens M, Hulscher M, Akkermans R, Hermens R, Grol R, Wollersheim H. The Team Climate Inventory: application in hospital teams and methodological considerations. Qual Saf Health Care. 2008 Aug;17(4):275-80. doi: 10.1136/qshc.2006.021543

36. Berendsen AJ, Benneker WH, Groenier KH, Schuling J, Grol RP, Meyboom-de Jong B. [DOC questionnaire – an instrument for measuring how GPs and medical specialists rate the quality of mutual collaboration (Dutch)]. Samenwerking tussen huisarts en specialist (Wat vinden de patiënten en dokters?). Houten: Bohn Stafleu van Loghum; 2008. p. 57-70. [Access date 2021 Feb 04]. Available from: https://www.rug.nl/research/portal/files/14515668/04emb_h4.pdf

37. Anderson NR, West MA. Measuring climate for work group innovation: development and validation of the team climate inventory. J Organ Behav. 1998 May;19(3):235-58.

38. Coleman EA, Mahoney E, Parry C. Assessing the quality of preparation for posthospital care from the patient's perspective: the care transitions measure. Med Care. 2005 Mar;43(3):246–55. doi: 10.1097/00005650-200503000-00007
