# Supplementary material for: Supporting care engagement in primary care; the development of a maturity matrix
Source: PLoS One. 2023 Jan 5;18(1):e0279542. doi: 10.1371/journal.pone.0279542 (PMC9815637; doi:10.1371/journal.pone.0279542)
Supplement: S2 File — (PDF) [file pone.0279542.s002.pdf]

S2 File. Elements in Maturity Matrix Care Engagement (MM-CE)

Personalised care	Shared decision making	Self-management	Patient as partner	Supportive means	Patient environment	Teamwork among healthcare professionals
		I support the handover of available patient information				
	I evaluate the decision making	I support the patient's solving of practical problems		I offer monitoring and lifestyle support	I refer to local information counters for specific support	
	I am working towards the joint decision	I support the patient's dealing with emotions	I involve the patient in the organisation of the general practice	I offer information and decision aids	I offer help in the search for social care	Our team follows joint team education
I tailor care to the patient	I ask about the patient's preferences and considerations	I support the patient's adherence to a healthy lifestyle	I see the patient as a co-producer of care	I offer support in setting healthcare goals	I support the informal caregivers	Our team shares the medical reporting
I put the whole patient in its own context	I discuss the options	I support the patient's monitoring of complaints/ symptoms	I have an open communication with the patient	I adapt the consultation to the patient	I involve the informal caregivers	Our team has regular team meetings
I see the patient as a unique person	I determine the problem together with the patient	I support the patient's correct intake and use of medication	I build a relationship of trust with the patient	I ensure open notes	I map the patient's personal network	Our team works according to a shared care vision

Two open questions:

1. *Name some strong aspects of the practice of yourself that contribute to the patient engagement.*
2. *Name some improvement options of the practice or yourself that can contribute to the patient engagement.*

Full instrument upon request available (in Dutch).
