# Supplementary material for: Supporting care engagement in primary care; the development of a maturity matrix
Source: PLoS One. 2023 Jan 5;18(1):e0279542. doi: 10.1371/journal.pone.0279542 (PMC9815637; doi:10.1371/journal.pone.0279542)
Supplement: Attachment — Submitted filename: Reviewer_feedback_PlosOne_20220528.docx [file pone.0279542.s003.docx]

Review of paper: Supporting care engagement in primary care; the development of a maturity matrix

General:

- Please go over the paper and correct any sentences that need grammar or sentence structure improvement. Also to include end brackets and other little details that have been missed.

Abstract

Background:

- First sentence is too vague. “Care engagement or active patients’ involvement in healthcare may contribute to the quality of primary care” – I think you need to be clear that increased engagement may improve quality of care.
- Second sentence doesn’t seem to link to first sentence – suggest need to amend to something like: maturity matrix considers organisational preconditions, and we developed and evaluated it… etc…

Background/Introduction

- Please change your language around older people – ‘elderly’ is not appropriate. Suggest you look at eg. From journals focused on research in older people for guidance: [When It Comes to Older Adults, Language Matters: Journal of the American Geriatrics Society Adopts Modified American Medical Association Style - Lundebjerg - 2017 - Journal of the American Geriatrics Society - Wiley Online Library](https://agsjournals.onlinelibrary.wiley.com/doi/full/10.1111/jgs.14941)
- Page 4, line 71 – person-centred care does not enable ‘compliance’ – in fact, that is the opposite of person-centred care. Suggest reframe.
- I wonder if the authors need to consider some papers on interprofessional collaborative care that focuses on team collaboration, where the person seeking healthcare is at the centre eg. [Interprofessional.Global](https://interprofessional.global/) and [Framework for action on interprofessional education & collaborative practice (who.int)](https://www.who.int/publications/i/item/framework-for-action-on-interprofessional-education-collaborative-practice) and [IPEC Core Competencies (ipecollaborative.org)](https://www.ipecollaborative.org/core-competencies) and [ASPPH | Interprofessional Collaborative Practice Model](https://aspph.org/teach-research/models/interprofessional-collaborative-practice/)
- I also wonder whether you need to bring in implementation science principles here – you state in page 5, line 77 “To translate this scientific information…’ – this is a field of science with underpinning core components. You are endeavouring to implement this into practice, so suggest you need to introduce what that means a bit more eg. An implementation framework that seems to fit what you are doing = CIFR [The Consolidated Framework for Implementation Research – Technical Assistance for users of the CFIR framework (cfirguide.org)](https://cfirguide.org/)
- Page 5, line 91 – the plural of matrixes is matrices
- Please review the grammar and language in the introduction – some tweaking needed eg. Sentence starting page 5, line 93 – think ‘a’ is messing after the first word ‘Such’, with that sentence being a bit clunky

Methods

Patient population - Broad inclusion criteria. Any exclusion criteria?

outcome measures - What about the resources needed to do this work as part of the feasibility and acceptability of undertaking this work? Ie. How long do people need to spend to do preparation, the actual filling out of the matrix, meeting with others etc..? Do they get back filled to do this work? Does it cost anything to do? Can they do it on their own or do they need get a facilitator in? Who pays for that? Etc..

Results

Table 1

- Patient participants – think we need to know their age, their education level, their socio-economic information, cultural background, other? – need a better picture of who they are to help us know from which perspective they are coming from;
- Patient organisations – are they not for profit? Government funded? How long have they been in operation? What size are they? Etc..
- Care professionals – age, education level, how long they have been working in total, and how long in the current role
- For all these participants – are they representative of their respective groups?

Table 2

- Need more info on the GPs, practice nurses etc… age, education undertaken, how long they have been working and how long they were in the current role.
- I wonder – what about receptionists being involved in something like this? They talk a lot with patients, and often know a lot about them…
- Is there any difference in these 2 practices that participated compared to the other 50 or so who had been sent invitations?

Discussion

- Suggest you start off with the core of what you found rather than the issue you are addressing.ie. Start with the sentence page 16 line 318 “This paper described the development as well as a first pilot…”
- Suggest your current first sentence needs reframing – “care engagement is thought to contribute to the quality of primary care”. You mean high care engagement = higher quality of care, so I suggest that is articulated.
- This discussion simply states what you did, and doesn’t relate it back into the existing literature – has anyone else done this kind of thing before? What did they find? How did your findings differ to what was done before? How does your work add to the knowledge base in this area?
- What about implementation aspects? This took considerable effort on the part of the research team to set up with the practices to implement – what would be needed for every general practice to do this? How would that even look? Is that even feasible? If this is an implementation study, need to consider what is needed to have this activity as part of ‘Business as Usual’ – as per my comment in the introduction, I strongly recommend you look up implementation science frameworks re: what is needed. A good starting point is reporting of implementation studies: Pinnock H, Barwick M, Carpenter CR, Eldridge S, Grandes G, Griffiths CJ, Rycroft-Malone J, Meissner P, Murray E, Patel A, et al. Standards for Reporting Implementation Studies (StaRI): explanation and elaboration document. BMJ Open. 2017;7:e013318. Things to consider – could practices do this on their own, or do they need a facilitator? How long would it take? Who would pay for backfill? What supports would be needed? What is needed to actually work through the practice changes identified from this process?
- One thing that also struck me – the groups came up with what they need to change, but struggled to make these changes. That must surely be somewhat anxious making for all participants – no one wants to fail. What is the impact of this inability to implement the changes they identified they needed? This can cause moral distress when care providers can’t deliver the best care they know they should.

Strengths and limitations

- You mention the low uptake to be a test practice – I suggest the authors response that it is due to limited interest is insufficient and would recommend reading the implementation science literature around the multitude of structural reasons behind limited uptake. This requires more comprehensive information – suggest asking a few of those who were approached but declined would also be important.

Practical and scientific implications

- Ah – now I see you bringing in other literature, thank you.
- I wonder – of the tool, what are the essential components to promote your goal – it looks like there are many components which takes a lot of time and input – is it all required? I think you do need to engage all practice team members – but do they need to do the same as the others? Should they look at things a bit differently, given their role and the skills they have are different? OR do you need to modify what you are considering so that you are only including those aspects relevant to all team members? What about receptionists? As I mentioned above, they know patients well – how can they be included? They are the first port of call to the practice, and can be barriers or enablers to patients feeling welcome and psychologically safe…
- You talk about raising enthusiasm alone is not enough – this leads into the implementation science approaches that need to be considered. Suggest more is needed here to support moving forward. The paragraph starting page 18 line 367 requires reworking – you wend in practice changes required by healthcare practices, then bring in the big issues around those who seek care and those factors that may limit their capability to engage ie. Health literacy, cognitive impairment etc… This needs more explanation. Right now, you have raised these topics, and they are very complex and you have just skimmed the surface.
- The last sentence starting line 381 is very long and convoluted - suggest reframing. Need to outline that the findings need to be used to adapt the current tool and test to ensure it meets the end user needs. Right now it is long, involved, not all team members can do all of it and it doesn’t lead to practice change. Sorry to be so blunt! But this is not a bad thing – it just means that this is where you have to focus your next steps – how can you make it include all key stakeholders? How can it be less time consuming – what can you leave out? What are the bits you simply must keep in? What are the essentials that could lead to real practice change? What supports are needed so practices can undertake practice change?

Conclusions

I think this tool requires significant adaptations to enable it to be used in practice, and that working out what is essential and how practice change can be supported are key.
