# Supplementary material for: Supporting care engagement in primary care; the development of a maturity matrix
Source: PLoS One. 2023 Jan 5;18(1):e0279542. doi: 10.1371/journal.pone.0279542 (PMC9815637; doi:10.1371/journal.pone.0279542)
Supplement: Attachment — Submitted filename: Response to reviewers second revision.docx [file pone.0279542.s005.docx]

**Point to point rebuttal regarding reviewers comments to the author**

Reviewer #1: Thank you for considering the feedback and making the changes you did. It is more comprehensive and reads much better. It is a good study and would be of interest to others researching and working to improve person-centred care delivery in general practice.

General: There is still some grammar and sentence structure improvement required.

- *Following your suggestion, we have had the manuscript checked by a text editor.*

Re: discussion;

You described the lessons learned using the seven aspects from Flottorp et al’s Implementation Framework – I am wondering whether this should be part of the results. I see this as an integral part of implementation evaluation – I don’t know that it fits in the discussion. If you agree, I think you would need to add that you use this framework to evaluate the implementation into your methods.

- *We understand the consideration, but the focus of our paper is on supporting care engagement in primary care for which we have developed a tool. We endorse the importance of taking implementation into account from the start. That is why we have* ***systematically*** *approached the development of the tool (see also the Medical Research Council guideline for the development of complex interventions) and have involved the local stakeholders from the start. Flottrop’s Implementation Framework was introduced after the evaluation of the study. We therefore don’t think it is methodologically sound to suggest this was already part of the original plan. However, we do value discussing our results in the context of this framework, as the framework makes the lessons learned more concrete.*

Reviewer #2: General:
• There is an inconsistent spelling of certain words throughout the manuscript (e.g., organisational / organizational, and Personalised / Personalized have been used interchangeably)
• Please consider copy-editing this manuscript. There are several grammar, spacing, spelling, and punctuation errors (the most significant being the overuse of unnecessary commas), alongside unclear antecedents and dangling modifiers. Appropriate quality copyediting should remove these errors.

I have listed some errors I found, but there are several more that need attention.

BOX: Grammatical suggestions form reviewer 2

**Abstract**
• Line 23 has a grammatical error (e.g. preconditions instead of preconditions)
**Introduction**
• The sentence spanning from lines 72-75 is too long (49 words). I know it is a quote, but Please consider splitting it into two or paraphrasing it into a shorter sentence.
• Line 76: It is not good practice to use unclear antecedents like ‘this’ to begin a sentence. It may be unclear what ‘this’ refers to. Consider re-writing the sentence to remove the unclear reference.
• Line 78: contains a dangling modifier (i.e. “To implement these insights on care engagement in a sustainable manner” does not appear to be modifying the subject). Sentence rearrangement should clarify this.
**Methods**
• The sentence spanning lines 189-191 needs rewriting for clarity. Some suggestions:
• Appropriate writing conventions require authors to write out any numbers under ten in full (e.g., ‘two’ instead of ‘2’)
• “of whom 2 implementation consultants” is missing a word.
• The sentence is too long and unclear.
• The sentence spanning lines 202-203 needs rewriting for clarity, and an end bracket.
• The sentence spanning lines 205-206 has a spelling error (where VS were). I suggest fixing.
**Results**
• Line 221: A total of 38 papers were considered relevant for the identification of the axis. This should be reworded, as there is overcomplexity and redundancy in the sentence formulation. (i.e., A total of 38 papers were considered relevant for axis identification). Sentence overcomplexity is a very common issue throughout this manuscript.
**Discussion**
• The sentence spanning lines 350-351 “This way we designed in a stepped way a target population centred tool.” Needs to be addressed for punctuation, grammar and clarity.
• There are several instances of incorrect use of conjunctions, The sentence on line 358 is an example, (kick-off session, facilitator session to support SMART improvement plans). Please add appropriate conjunctions (e.g., ‘and’ where appropriate).
• There are several instances of incorrect word spacing throughout the manuscript. The sentence on line 359 is an example.
• There are several instances of incorrect, or lacking, punctuation use. The sentence on lines 365-368 is an example (Although there are recent studies on efforts to increase patient engagement, to our knowledge there is no existing tool…) I suggest to add in comma handles where appropriate to this sentence. Please also review the manuscript to remove excessive or add lacking punctuation. The sentence on lines 398-400 is also an example of lacking punctuation.

- *Thank you for carefully reading our contribution. We have adapted the passages you have designated. In addition, we also had the text reviewed by a professional editor, as you suggested.*
